# Severe Sepsis Secondary to Listeriosis in a 73-Year-Old Male With Multiple Comorbidities

**DOI:** 10.7759/cureus.76915

**Published:** 2025-01-04

**Authors:** Samin Rahman, Kanza Shamim, Shadab Ahmed

**Affiliations:** 1 Internal Medicine, Nassau University Medical Center, East Meadow, USA; 2 Infectious Disease, Nassau University Medical Center, East Meadow, USA

**Keywords:** bacteremia, listeria monocytogenes, listeriosis, sepsis, septic shock

## Abstract

This case discusses a 73-year-old male who presented to the emergency department with fever, diarrhea, weakness, and altered mental status. Initial blood cultures grew *Corynebacterium; *however, cultures were later corrected to *Listeria monocytogenes.* The patient ultimately passed away from complications of sepsis secondary to listeriosis*.*

## Introduction

*Listeria monocytogenes* is an intracellular gram-positive rod often transmitted via foods such as unpasteurized dairy products and cold deli meats. It can cause a severe infection in at-risk populations, including the elderly, neonates, and immunocompromised patients [1]. In all the aforementioned patient populations, the incidence of sepsis secondary to *Listeria* infection is about 46% [2].

This case discusses a 73-year-old male with multiple comorbidities who was admitted for sepsis and bacteremia secondary to *Listeria monocytogenes* infection.

## Case presentation

History

The patient was a 73-year-old male with a past medical history of coronary artery disease, heart failure with reduced ejection fraction (25-30%), previous myocardial infarction status post-percutaneous coronary intervention, hypertension, hyperlipidemia, type 2 diabetes mellitus, gout, and prostate cancer status post prostatectomy who initially presented to the emergency department with chief complaints of weakness and altered mental status for two days prior to coming to the hospital. The patient also endorsed intermittent subjective fevers, diarrhea, and loss of appetite. The patient was noted to have a history of regularly consuming deli meats.

Physical exam

On initial vitals, the patient was afebrile. However, he was tachycardic with a heart rate of 102. Intake blood pressure was 123/79, and the patient was saturating 98% on room air with a respiratory rate of 18 and GCS of 15. On initial physical examination, the patient did not appear acutely ill and did not appear to be in any acute distress. However, the following day, the patient appeared acutely ill on examination, with increased work of breathing and lethargy. At that time, he was noted to have a temperature of 103.6℉. Table 1 shows the lab results.

**Table 1:** Selected lab values WBC: white blood cells, HBG: hemoglobin, PLT: platelet, ESR: erythrocyte sedimentation rate, Na: sodium, K: potassium, ALT: alanine aminotransferase, AST: aspartate aminotransferase, CRP: C-reactive protein

Parameters	Patient values	Reference range
WBC	13.66 x 10^3/uL	4.5-11.0 x 10^3/uL
HGB	16.7 g/dL	13.5-18.0
PLT	158 k/mm^3	150-450
% neutrophil	89.6%	38.9-75.1%
ESR	33 mm/Hr	0-20 mm/Hr
Na	132 mmol/L	136-145 mmol/L
K	4.6 mmol/L	3.5-5.1 mmol/L
Bicarbonate	16 mmol/L	20-31 mmol/L
Urea nitrogen	35 mg/dL	9-23 mg/dL
Creatinine	2.0 mg/dL	0.7-1.3 mg/dL
ALT	43 u/L	7-40 u/L
AST	38 u/L	13-40 u/L
Troponin I	61.54 ng/L→ 70.41	00.00-53.53 ng/L
Lactate	1.8 mmol/l	0.5-1.6 mmol/l
CRP	10.7 mg/dL	0.0-0.9 mg/dL
D-dimer	1.76 ug/ml	<0.5 ug/ml

Hospital course

**Figure 1:**
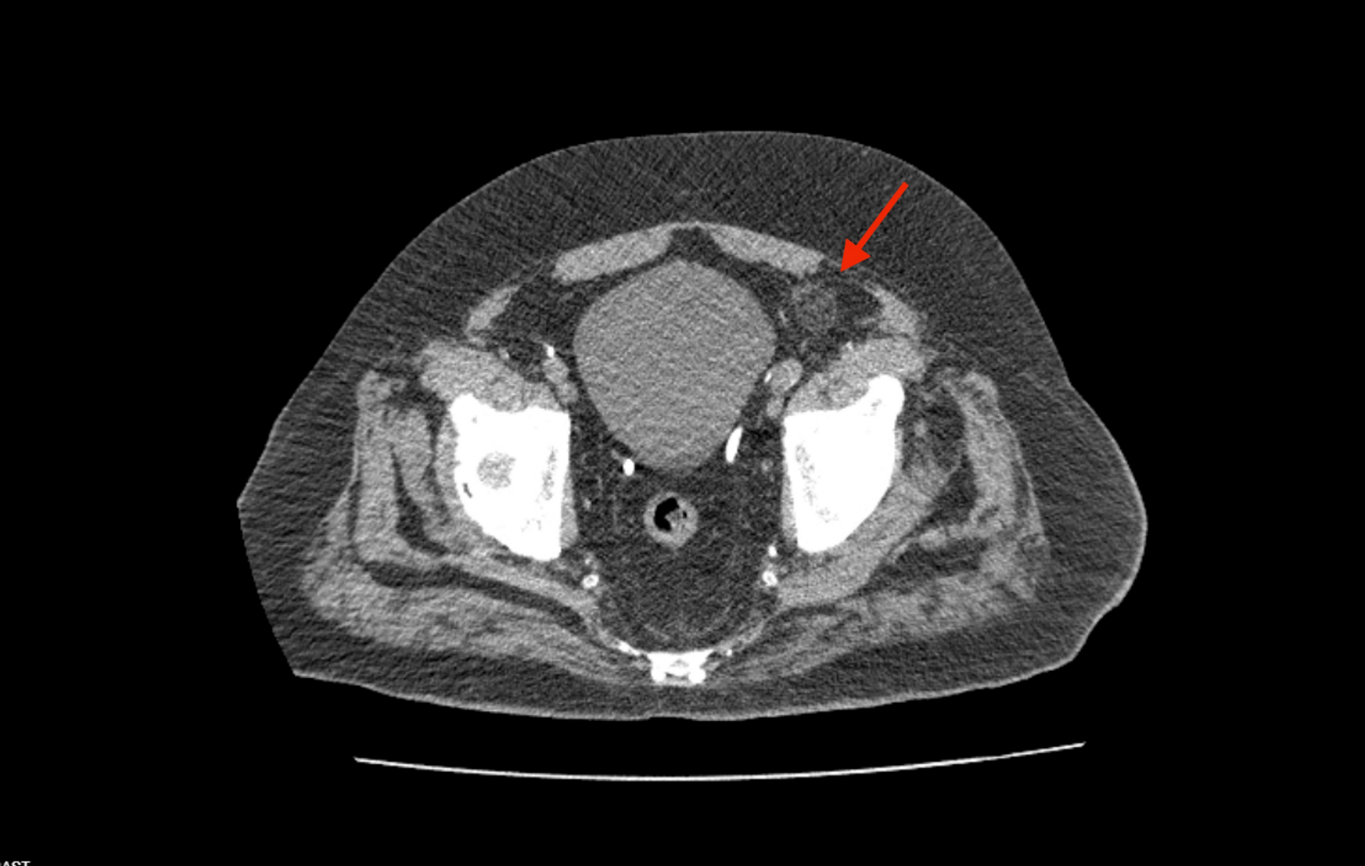
CT abdomen and pelvis demonstrating sigmoid epiploic appendagitis CT: computed tomography

**Figure 2:**
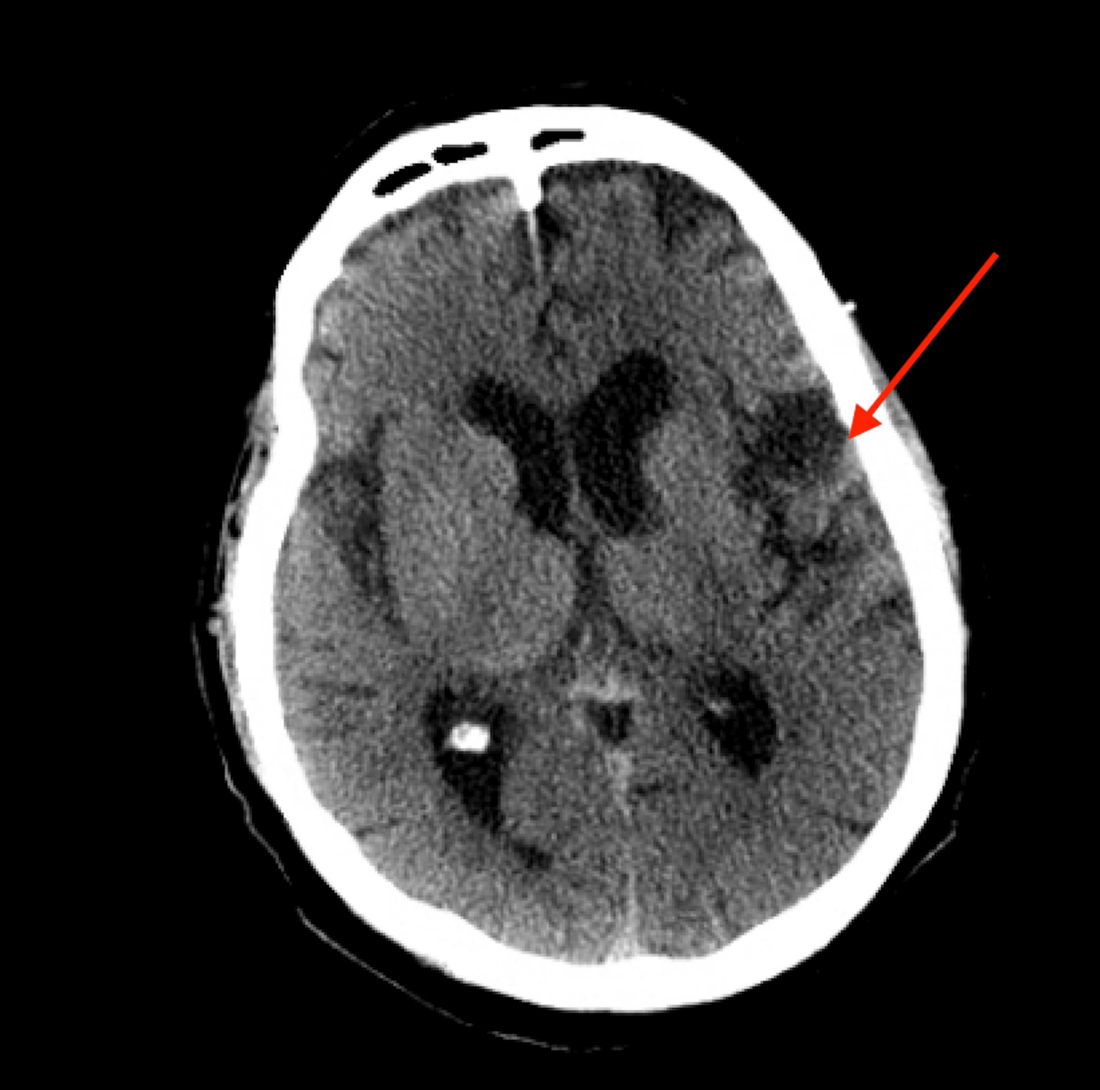
CT head showing left parietal lobe encephalomalacia CT: computed tomography

The patient was initially admitted to the telemetry service in the elevated troponins setting. CT scans done on admission showed sigmoid epiploic appendagitis on abdominal CT (Figure 1) and encephalomalacia on CT brain (Figure 2). The patient was started on ceftriaxone and metronidazole, and fluid resuscitation was initiated. On the night of admission, the patient deteriorated further clinically and had high fevers with a max temperature of 103.6℉ and had a seizure; subsequently, he was intubated and transferred to the medical ICU. Blood cultures drawn on admission initially reported *Corynebacterium*. In addition, urine cultures taken at admission were positive for *Proteus mirabilis* and *Klebsiella pneumoniae*; however, urinalysis done on admission did not have any significant WBCs. The patient’s antibiotic coverage was escalated to piperacillin/tazobactam 4.5 mg every eight hours instead of ceftriaxone in the setting of persistent fevers. A right internal jugular central line was placed the following day, and the patient was started on vasopressors. The infectious disease team recommended lumbar puncture to rule out additional sources of infection; however, the patient was too clinically unstable for the procedure. Blood culture speciation was later corrected to *Listeria monocytogenes*, and the patient’s antibiotic therapy was changed from piperacillin/tazobactam to ampicillin 2 g every eight hours, and vancomycin by level was continued based upon serum creatinine. After discussing the blood culture results with the patient’s family at the bedside, the patient's family endorsed that the patient would frequently consume sandwiches from the deli. Despite the change in antibiotic regimen, the patient continued to spike fevers, so micafungin was empirically added for antifungal coverage. The patient continued getting the aforementioned treatments; however, on hospital course day 6, given the patient’s worsening condition and poor prognosis requiring full ventilator support and increasing vasopressor requirements, the patient's family stated they wished for the patient to be made comfortable and to be terminally extubated and for the cessation of antibiotic treatment, and the patient ultimately passed on day 7 of hospital course.

## Discussion

Depending on the age group affected, *Listeria monocytogenes* can have a wide spectrum of presentations. As previously mentioned, particular groups at risk include infants, immunocompromised, and elderly patients [1-3]. Mortality secondary to *Listeria* infection is relatively uncommon. According to the CDC, roughly about 260 people die from *Listeria* infection annually in the United States. The mortality rate for *Listeria* infection varies from roughly 10-30% [4]. Given that this patient had multiple medical comorbidities, including heart failure with reduced ejection fraction, coronary artery disease, and a prior history of cancer, his mortality secondary to sepsis was already very high.

In this case, the diagnosis of listeriosis was not confirmed until blood culture results were corrected and speciation was available to the ICU and infectious disease teams. However, as per sepsis treatment protocols, the patient received adequate fluid resuscitation and timely broad-spectrum antibiotic coverage on admission. In addition, the information that the patient frequently ate cold-cut deli meats came upon more specific questioning after the blood culture results were reported. This delay in microbiological identification likely also contributed to the patient’s death, as the appropriate antibiotic therapy could not be given earlier in the hospital course.

In addition, CT imaging on admission showed evidence of sigmoid epiploic appendagitis. Sigmoid epiploic appendagitis is inflammation of the epiploic appendages of the colon and is a relatively uncommon cause of abdominal pain. It typically occurs in obese men in their fourth to fifth decade of life, occurring in about 8.8 in every 1 million patients. [5] In this patient, the sigmoid epiploic appendagitis was likely secondary to *Listeria* without other possible causes. Listeriosis can be classified generally into two categories: non-invasive, which typically affects immunocompetent individuals with gastroenteritis, and invasive, which is associated with septicemia in immunocompromised or at-risk individuals. The mortality associated with the latter group is about 20-30% [6]. Given that this patient met sepsis criteria and had multiple other prior medical issues, his mortality from *Listeria* was likely much higher. In this case, once *Listeria* was identified, ampicillin was the antibiotic of choice. However, other usual antibiotic therapies indicated in listeriosis include intravenous penicillin G or trimethoprim-sulfamethoxazole if the patient has a history of penicillin allergy [1].

In light of the large-scale *Listeria monocytogenes* outbreak across multiple states linked to deli meats in recent weeks, as of September 23, 2024, the CDC has reported 59 confirmed cases of listeriosis, with 59 of the aforementioned patients hospitalized. Of the 59 hospitalized patients, there have been 10 deaths confirmed thus far [3]. The median age of reported cases was 74, with more than 75% reported in White patients. In addition to deli meats, additional foods have been linked to the outbreak, including 71 products sold under Boar’s Head brand deli meats, which amounts to approximately 7 million pounds of meat and poultry products, showcasing the extent of this outbreak [7].

## Conclusions

It is important to highlight this case, as listeriosis can often be overlooked as a potential source of infection in elderly patients admitted with gastrointestinal symptoms. Given the scale of the ongoing *Listeria* outbreak, it is entirely plausible that the number of cases is being underreported, given generally lower clinical suspicion. However, as seen in this case and the setting of the current outbreak especially, listeriosis cannot be overlooked. It can often have fatal outcomes for patients in at-risk groups and requires early and targeted intervention.
